# Supplementary material for: Biased cytochrome P450-mediated metabolism via small-molecule ligands binding P450 oxidoreductase
Source: Nat Commun. 2021 Apr 15;12:2260. doi: 10.1038/s41467-021-22562-w (PMC8050233; doi:10.1038/s41467-021-22562-w)
Supplement: Supplementary file 1 — Supplementary Information [file 41467_2021_22562_MOESM1_ESM.pdf]

Supplementary Materials for

Biased cytochrome P450-mediated metabolism via small-molecule ligands binding P450 oxidoreductase

Authors

Simon Bo Jensen[1], Sara Thodberg[2,3,4], Shaheena Parween[5,6], Matias E. Moses[1], Cecilie C. Hansen[2,3,4], Johannes Thomsen[1], Magnus B. Sletfjerding[1], Camilla Knudsen[2,3,4], Rita Del Giudice[2,3,4], Philip M. Lund[1], Patricia R. Castaño[5,6], Yanet G. Bustamante[1], Maria Natalia Rojas Velazquez[5,6], Flemming Steen Jørgensen[7], Amit V. Pandey[5,6], Tomas Laursen[2,3,4], Birger Lindberg Møller[2,3,4,8] & Nikos S. Hatzakis[1,9*]

Affiliations

[1] Department of Chemistry & Nanoscience Centre, University of Copenhagen, Universitetsparken 5, DK-2100 Copenhagen Ø, Denmark.

[2] Plant Biochemistry Laboratory, Department of Plant and Environmental Sciences, University of Copenhagen, Thorvaldsensvej 40, DK-1871 Frederiksberg C, Copenhagen, Denmark.

[3] Center for Synthetic Biology, Thorvaldsensvej 40, DK-1871, Frederiksberg C, Copenhagen, Denmark.

[4] VILLUM Research Center "Plant Plasticity", Thorvaldsensvej 40, DK-1871, Frederiksberg C, Copenhagen, Denmark.

[5] Pediatric Endocrinology, Diabetology, and Metabolism, University Children's Hospital, Freiburgstrasse 15, CH-3010 Bern, Switzerland.

[6] Department of Biomedical Research, University of Bern, Murtenstrasse 35, CH-3008 Bern, Switzerland.

[7] Department of Drug Design and Pharmacology, University of Copenhagen, Jagtvej 160, DK-2100 Copenhagen Ø, Denmark.

[8] Carlsberg Research Laboratory, J. C. Jacobsen Gade, DK-1799 Copenhagen V, Denmark.

[9] Novo Nordisk Foundation Centre for Protein Research, Faculty of Health and Medical Sciences, University of Copenhagen, Blegdamsvej 3B, DK-2200 Copenhagen N, Denmark.

* Corresponding author. E-mail: hatzakis@chem.ku.dk

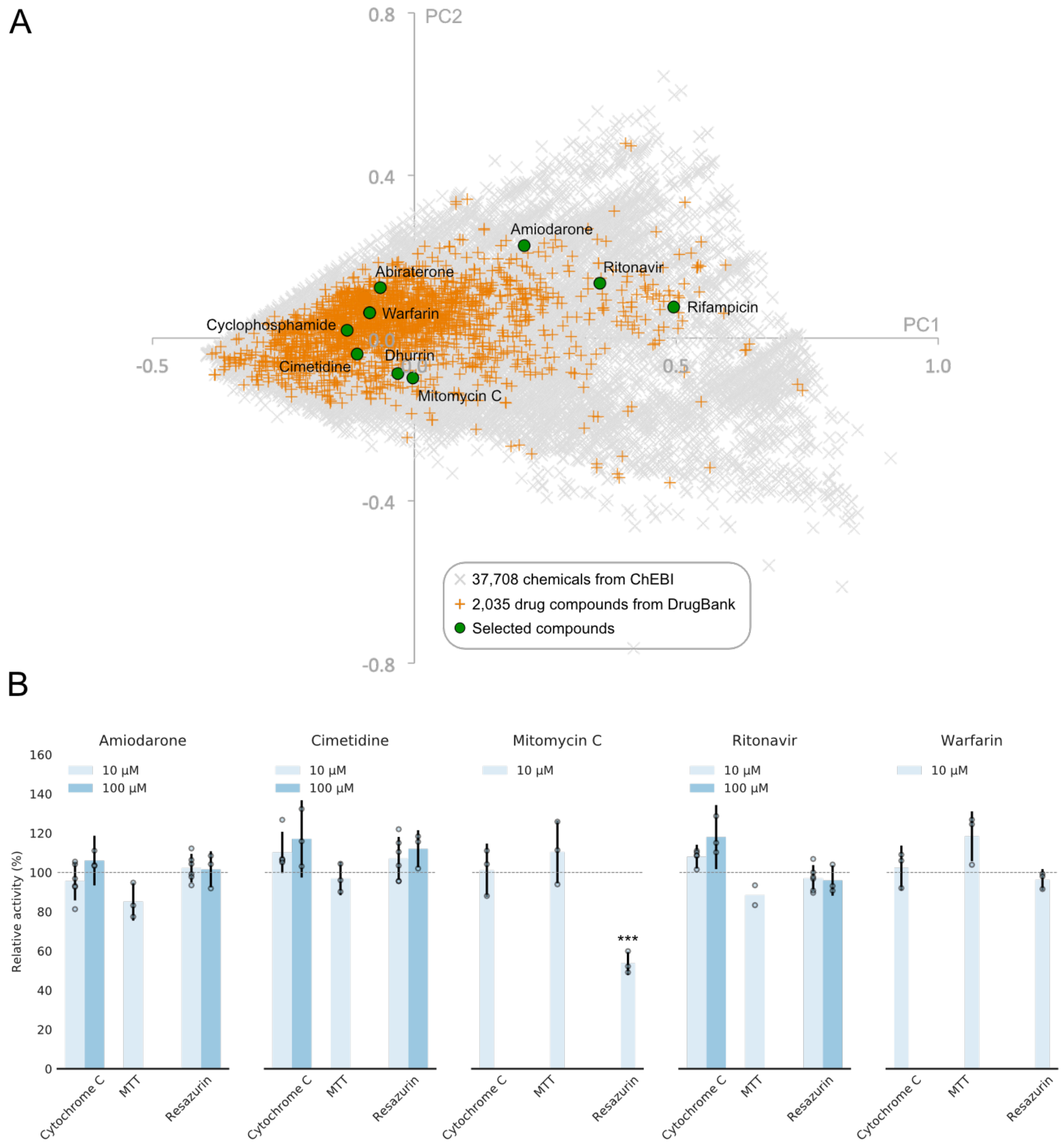

Supplementary Fig. 1. A) Plot of the two first principal components (PC1 and PC2) of the combined ChEBI and Drugbank datasets. Compounds from the ChEBI and Drugbank datasets shown as grey and orange symbols, respectively. Green circles highlight compounds studied experimentally in this work B) *In vitro* assays of small-molecule ligands showing weak or no effects on human POR proteoliposome activity. Bar charts display activity normalized to DMSO controls in the Cyt*c*, MTT and RS assay, respectively. Mitomycin C acts as a specific inhibitor/antagonist towards RS reduction (54±6 % of control), but is directly reduced by POR [1]. The remaining compounds have no statistically significant effect on POR function at the tested concentrations. Therefore, further studies at higher concentrations, or single molecule level or on cells were not performed. Error bars represent mean ± SD of independent replicates normalized to

controls with error propagation (n=2-6; see Supplementary Table 3 for exact value of n for each experimental condition). Overlapping data points appear shaded. The level of significance determined by one-way ANOVA and Tukey's HSD test correcting for multiple comparisons is marked by asterisk symbols (* $p<0.05$; ** $p<0.01$; *** $p<0.005$; see Supplementary methods for details).

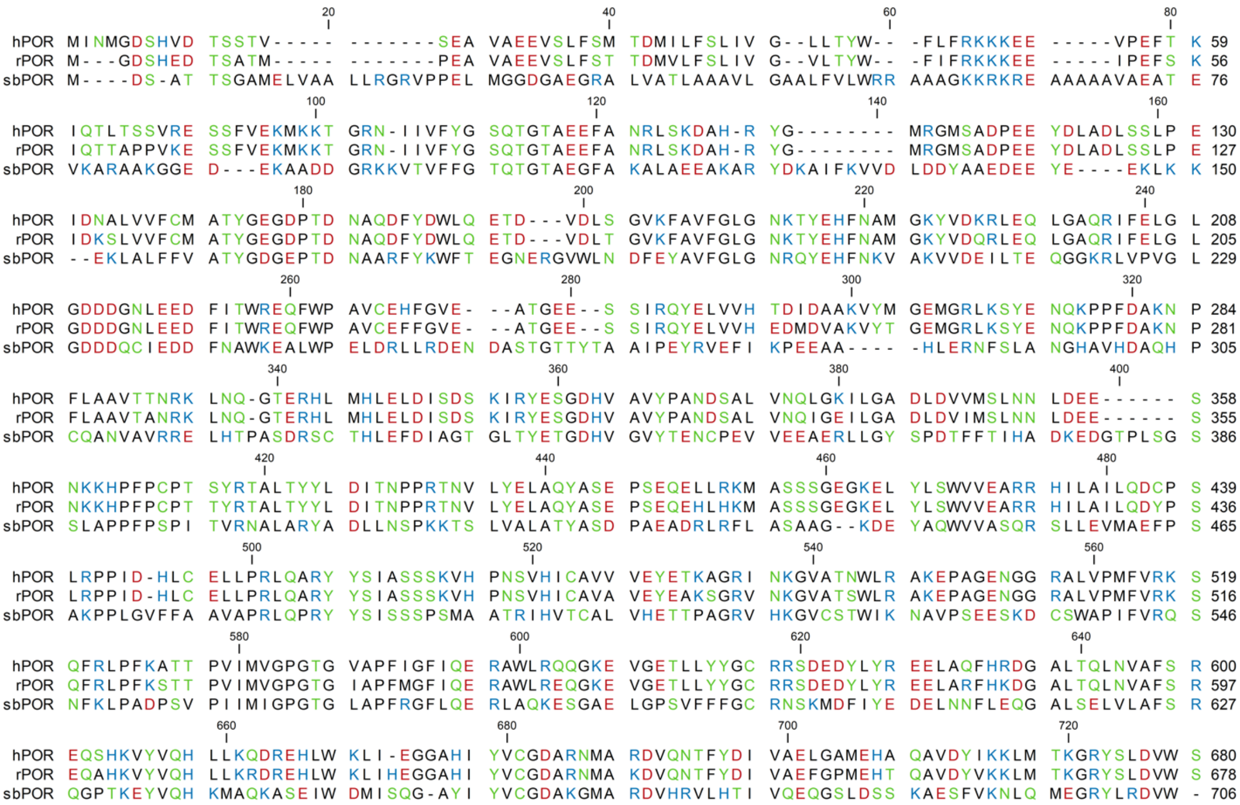

Supplementary Fig. 2. Sequence alignment between human, rat and sorghum bicolor POR. The POR protein sequences were obtained from NCBI protein database and aligned using ClustalW (www.ebi.ac.uk/clustalw) and prepared for visualization with CLC Protein Workbench (CLC Bio, Aarhus, Denmark). We used the amino acid sequences of *Homo sapiens* (NCBI: NP_000932.3), *Rattus norvegicus* (NCBI: NP_113764.1), *and Sorghum bicolor* (NCBI: XP_002444097.1). The human and rat POR are almost identical (>94% sequence identity), while human and sorghum POR shares 38% sequence identity. Core structural elements of flavin and NADPH binding are conserved across species as we have shown previously [2,3]. A large number of disease-causing mutations in POR have been identified, and several of the residues involved in docking of compounds tested in this manuscript are located at or around these places [4].

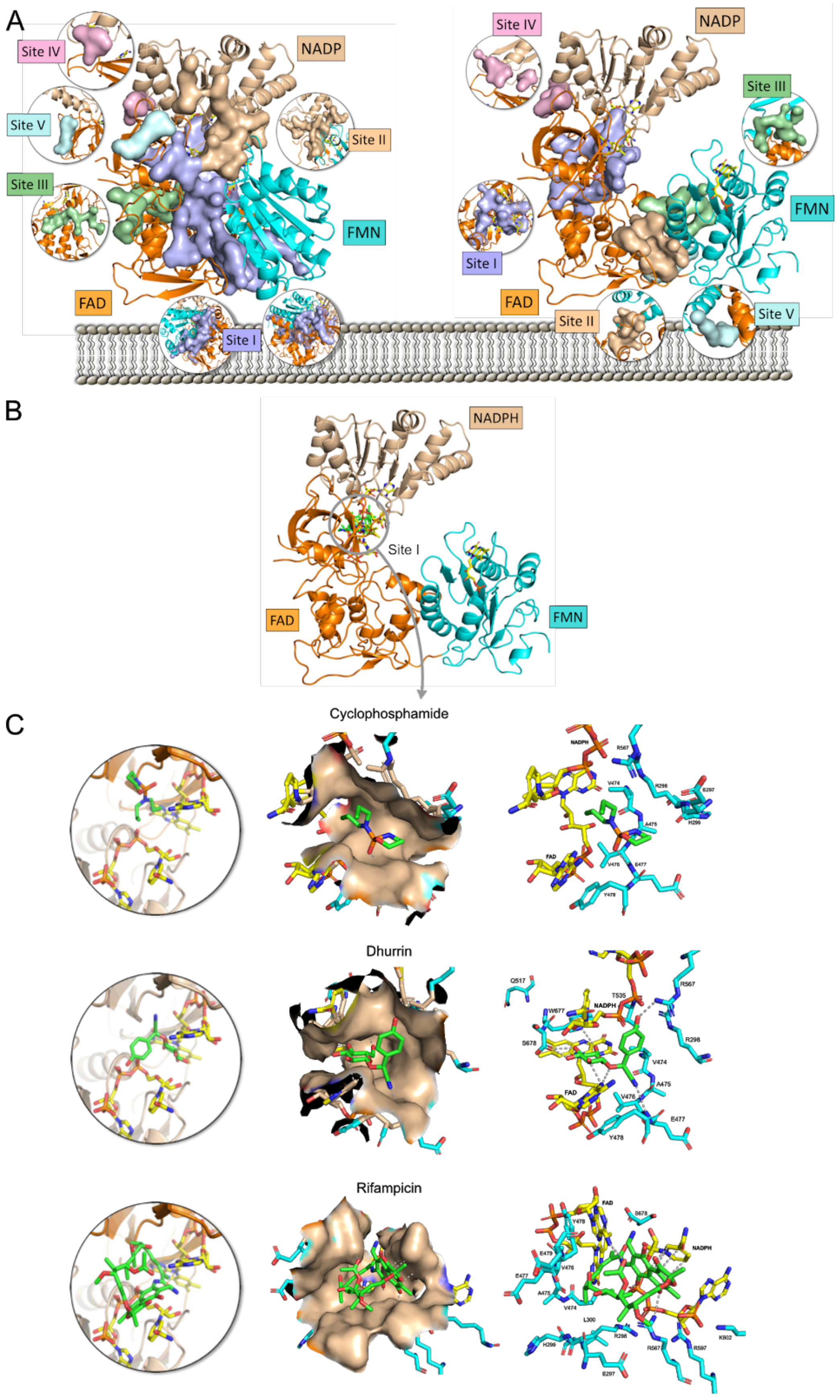
A
Site IV
NADP
Site V
Site II
Site III
FMN
FAD
Site I
Site IV
NADP
Site III
FMN
Site I
FAD
Site II
Site V
B
NADPH
Site I
FAD
FMN
C
Cyclophosphamide
Dhurrin
Rifampicin

Supplementary Fig. 3. Potential ligand binding sites and docking of small-molecule ligands on rat POR. A) SiteMap analysis on human POR in a compact conformation (PDB 3QE2; left) and rat POR in an extended conformation (PDB 3ES9; right) identifying five possible ligand binding sites (Sites I-V) on both isoforms. Sites I-III display SiteScore and Dscore values indicating that ligands may bind to these sites with sub-micromolar affinity (see Supplementary Table 1). Note, Sites I-V on human POR do not completely align with Sites I-V on rat POR. B) Binding of small-molecule ligands on rat POR in an extended conformation (PDB 3ES9). Ligands are displayed in green, while cofactors are displayed in yellow. C) Predicted binding of cyclophosphamide, dhurrin and rifampicin in Site I. All amino acid residues (blue) and cofactors (yellow) within 5 Å from the respective ligands are displayed. Cyclophosphamide is predicted to form H-bonds to A475 (2.8 Å) and E477 (3.1 Å). Dhurrin is predicted to form H-bonds to E477 (3.5 Å), R567 (2.8 Å), W677 (3.3 Å), S678 (2.7 Å and 2.8 Å), NADPH (2.6 Å) and FAD (2.9 Å, 3.0 Å, 3.1 Å). Rifampicin is predicted to form H-bonds to R567 (3.0 Å), NADPH (2.9 Å and 3.0 Å) and FAD (3.3 Å), and pi-pi interactions with NADPH (3.7-4.3 Å). See Supplementary Table 2 for predicted binding energies.

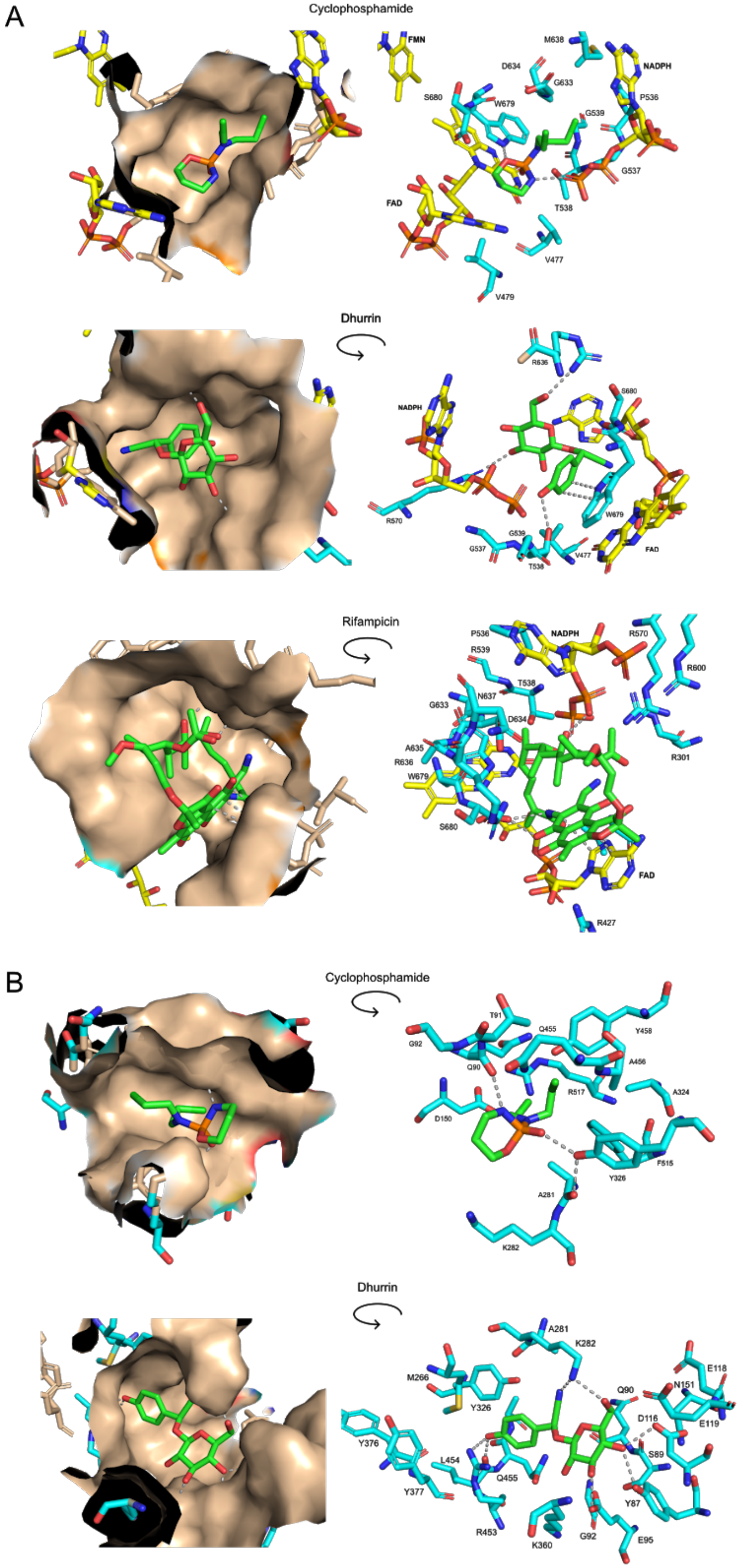
A
Cyclophosphamide
FMN
M638
NADPH
D634
G633
S680
W679
G539
P536
G537
FAD
T538
V477
V479
Dhurrin
R636
S680
NADPH
R570
W679
G539
G537
T538
V477
FAD
Rifampicin
P536
NADPH
R570
R539
R600
T538
G633
N637
D634
A635
R636
W679
R301
S680
FAD
R427
B
Cyclophosphamide
T91
Q455
Y458
G92
Q90
A456
R517
A324
D150
F515
Y326
A281
K282
Dhurrin
A281
K282
M266
Y326
Q90
E118
N151
D116
E119
Y376
L454
S89
Q455
Y87
Y377
R453
K360
G92
E95

Supplementary Fig. 4. Predicted docking conformations of small-molecule ligands on human POR (PDB 3QE2) in Site Ia (A) and Site Ib (B). All amino acid residues (blue) and cofactors (yellow) within 5 Å from the respective ligands (green) are displayed. In Site Ia, Cyclophosphamide appears to bind in a cavity with the chlorines tightly fitting into a groove partly formed by NADPH. It is predicted to form H-bonds to W679 (3.3 Å) and NADPH (2.6 Å). Dhurrin also binds in a cavity between NADPH and FAD with predicted H-bonds to T538 (2.9 Å), R570 (3.2 Å), R636 (2.9 Å) and pi-pi interactions with W679 (3.6-4.2 Å). Rifampicin is predicted to form pi-pi interactions with FAD (3.6-4.3 Å), a H-bond to S680 (2.8 Å) and two H-bonds to NADPH (2.4 Å and 3.2 Å). Notably, amino acid residues G539 and R600 which are within 5 Å from the three ligands in Site Ia are both associated with POR deficiency. Pathogenic mutations G539R and R600W both cause disorder of sexual development due to low production of sex steroids [2,5]. In Site Ib, cyclophosphamide is predicted to form H-bonds to A475 (2.8 Å) and E477 (3.1 Å). Dhurrin is predicted to form H-bonds to E477 (3.5 Å), R567 (2.8 Å), W677 (3.3 Å), S678 (2.7 Å and 2.8 Å), NADPH (2.6 Å) and FAD (2.9 Å, 3.0 Å, 3.1 Å), and rifampicin is predicted to form H-bonds to R567 (3.0 Å), NADPH (2.9 Å and 3.0 Å) and FAD (3.3 Å), and pi-pi interactions with NADPH (3.7-4.3 Å).

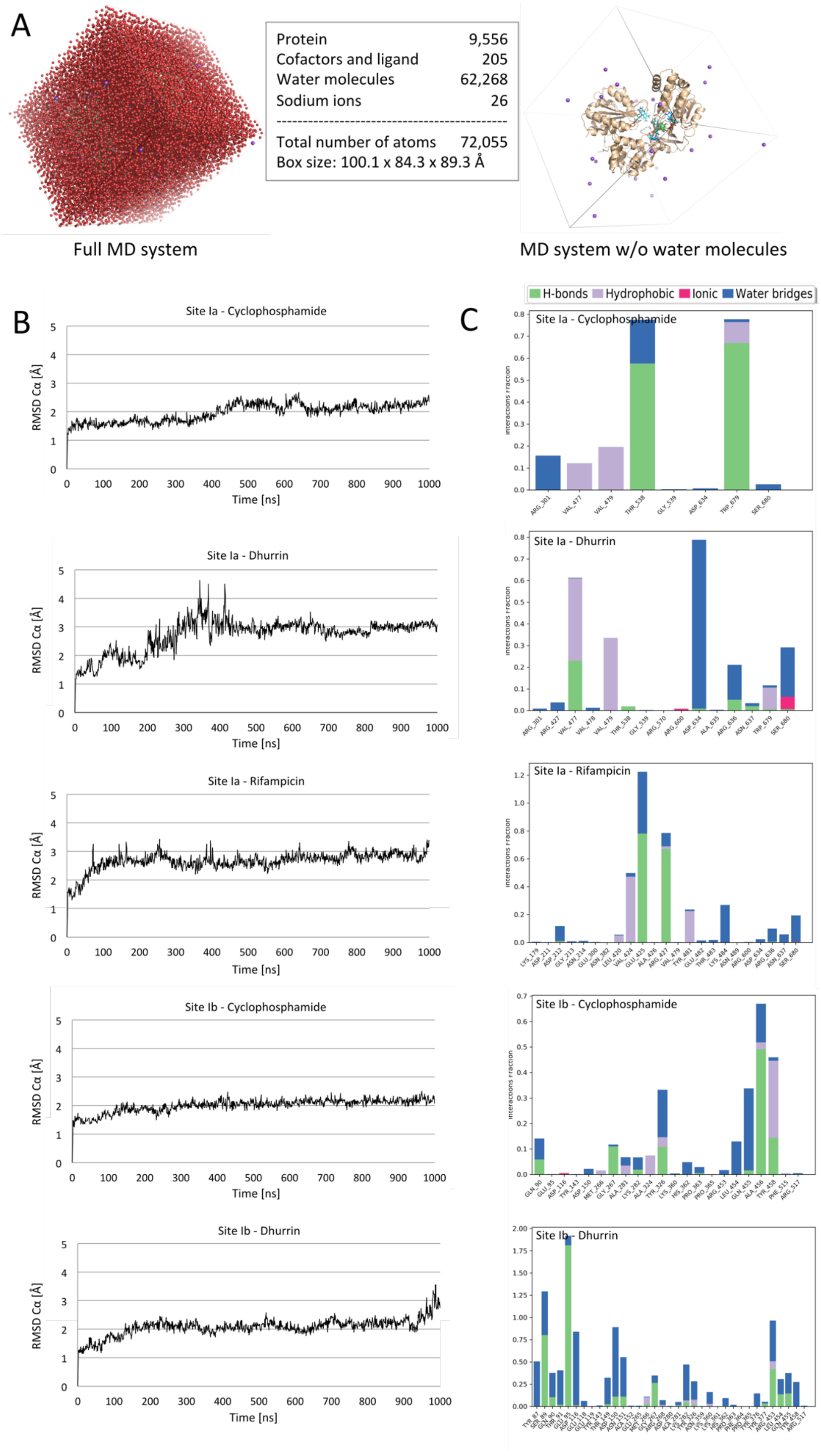
A
Protein 9,556
Cofactors and ligand 205
Water molecules 62,268
Sodium ions 26
Total number of atoms 72,055
Box size: 100.1 x 84.3 x 89.3 Å
Full MD system
MD system w/o water molecules
B
Site Ia - Cyclophosphamide
Site Ia - Dhurrin
Site Ia - Rifampicin
Site Ib - Cyclophosphamide
Site Ib - Dhurrin
RMSD Cα [Å]
Time [ns]
C
H-bonds
Hydrophobic
Ionic
Water bridges
Site Ia - Cyclophosphamide
Site Ia - Dhurrin
Site Ia - Rifampicin
Site Ib - Cyclophosphamide
Site Ib - Dhurrin
Interactions Fraction

Supplementary Fig. 5. Key parameters for MD simulations of the top scoring conformations obtained by docking on human POR (PDB 3QE2) and displayed in Supplementary Fig 3. A) 3D representation of one of the systems subjected to MD simulations primarily illustrating the water molecules populating the orthorhombic box (left), the same system with the water molecules undisplayed (right, same coloring as on Supplementary Fig 3, purple spheres are sodium ions, and numerical details on the system (middle). B) Cα root-mean-squares-deviations (RMSD) for the five protein-ligand complexes shown in Supplementary Fig 3 showing that the complexes remain stable during the MD simulations after an initial equilibration phase. C) Primary protein-ligand contacts encountered during the MD simulations for the five complexes (H-bonds are green, water bridges are purple, ionic contacts are pink and water bridges are blue).

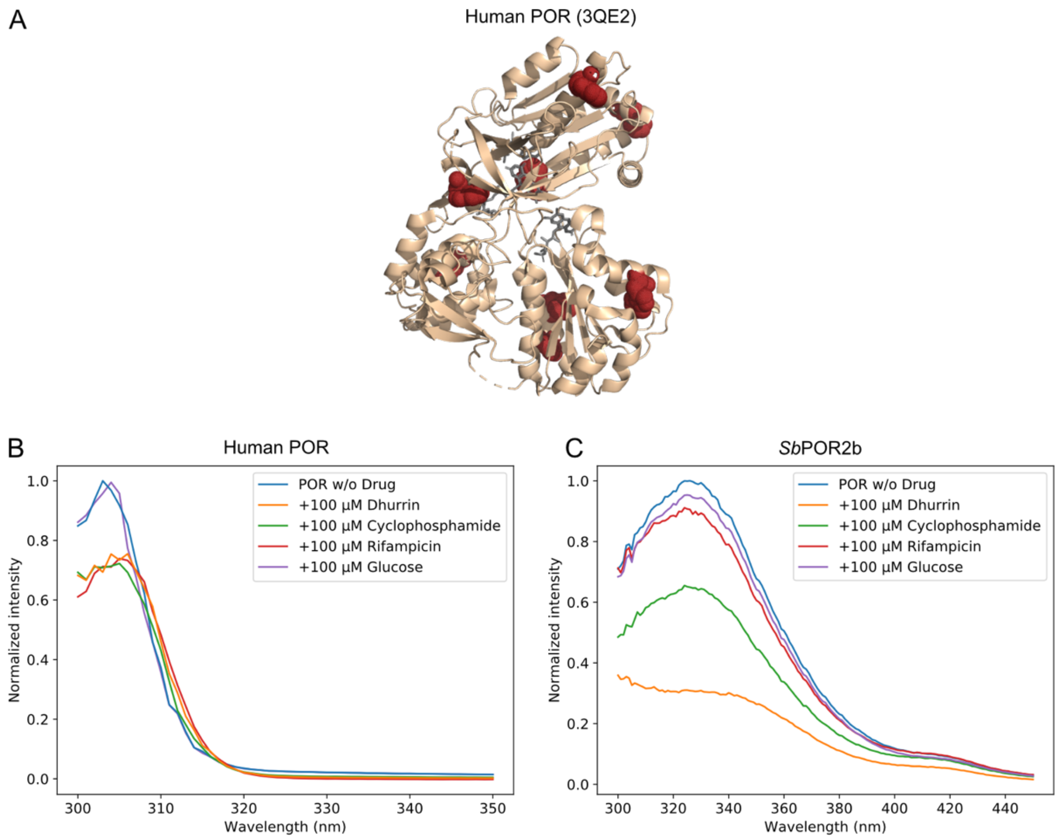

Supplementary Fig. 6. A) Structure of human POR (PDB 3QE2) highlighting tryptophan positions (red spheres). Flavin cofactors are displayed in grey. B+C) Intrinsic fluorescence quenching of human POR in microsomes (B) and *Sb*POR2b in detergent (C) by ligands (dhurrin, cyclophosphamide, and rifampicin). Glucose was used as a negative control. Fluorescence of POR showed decreased intensity after addition of 100uM of each of the ligands confirming their binding on POR. All spectra are corrected for background (i.e. buffer and ligand contribution).

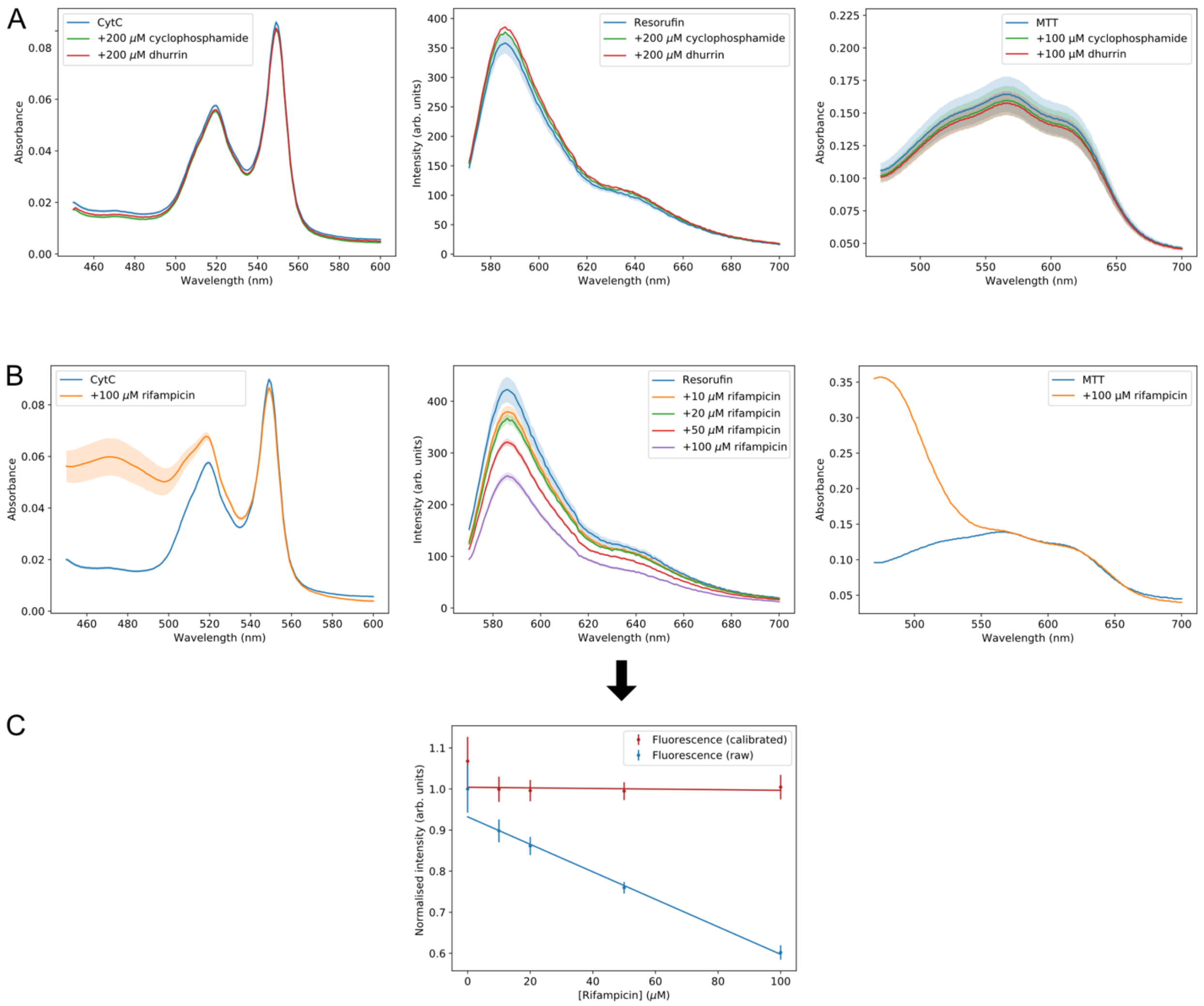

Supplementary Fig. 7. Control spectra and calibration of small-molecule ligands on Cytc, resorufin and MTT spectral properties. A) Control spectra showing that neither cyclophosphamide nor dhurrin induce any photophysical effects on Cyt*c* absorbance, resorufin emission nor MTT absorbance. B) Control spectra of rifampicin on Cyt*c* absorbance, resorufin emission (540 nm excitation) and MTT absorbance. Rifampicin does not significantly affect the absorbance readout of Cyt*c* nor MTT at the relevant wavelengths (550 nm and 610 nm, respectively), however resorufin emission is quenched by rifampicin in a concentration dependent manner (10-100 μM rifampicin). C) Calibration curve showing a linear correlation between rifampicin concentration and fluorescence intensity of resorufin extracted at the 585 nm emission peak (blue curve). To account for fluorescence quenching in the presence of rifampicin, a calibration was performed (red curve). Error bars represent mean ± SEM of three independent measurements. All rifampicin measurements presented in this study have been calibrated accordingly (RS assay only). A-B) Each spectrum represents the mean ± SEM of three independent measurements (shaded area).

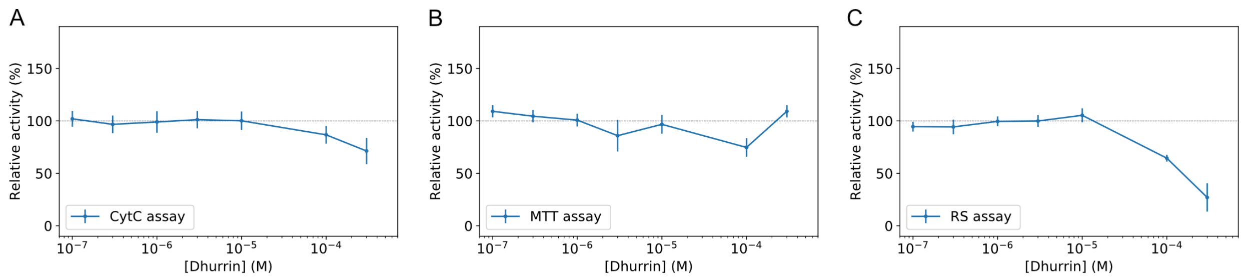

Supplementary Fig 8. Dose-response curves of dhurrin addition on the capacity of hPOR in microsomes to reduce CytC (A), MTT (B), Resazurin (C). Error bars represent the mean ± SD of three independent measurements.

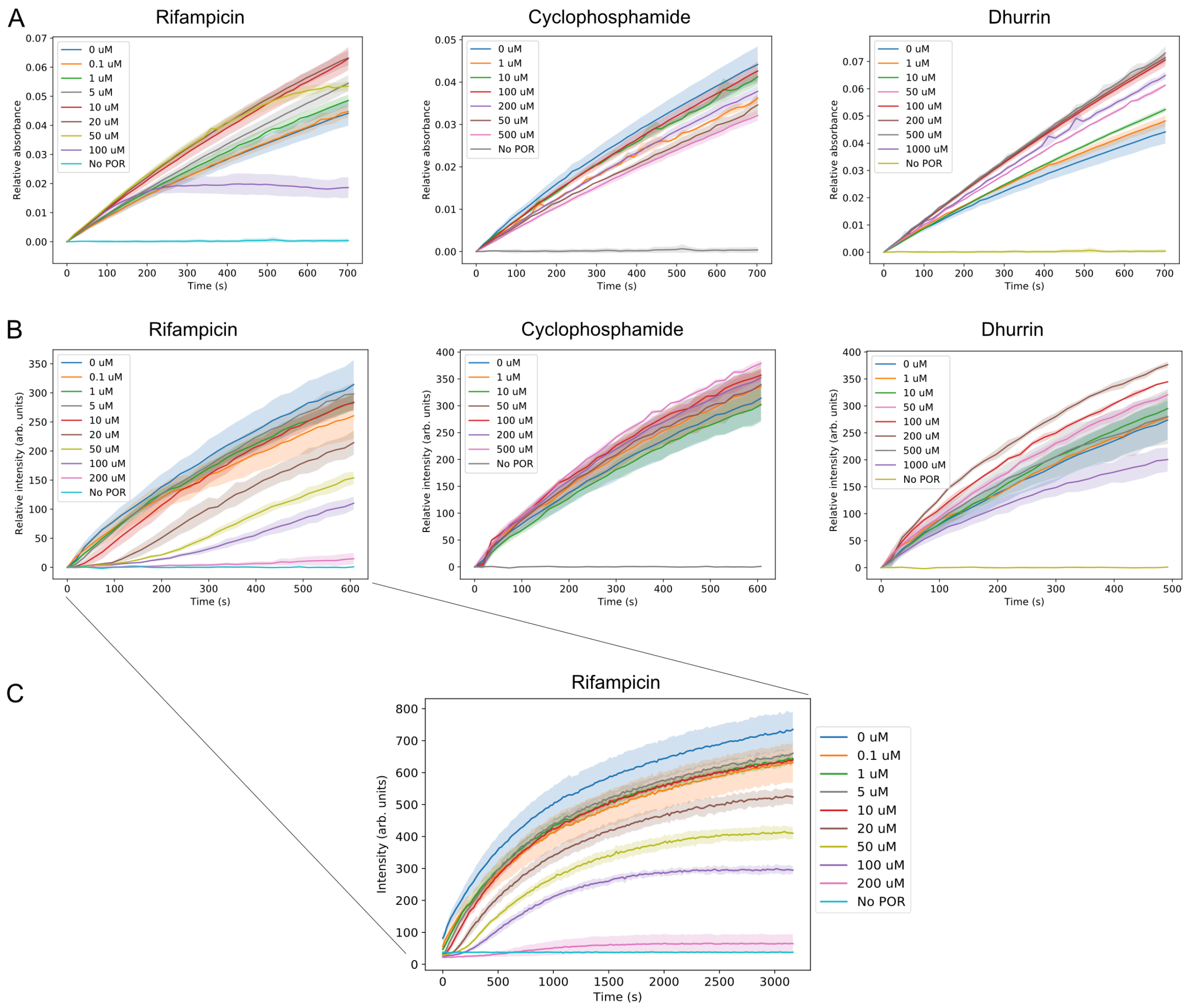

Supplementary Fig. 9. Raw activity traces of an experiment on *Sb*POR2b proteoliposomes in A) Cyt*c* assay and B) RS assay. The linear region of each trace was used to quantify activity. Rifampicin traces are calibrated according to Supplementary Fig 7 and the activity is extracted after the lag phase (discussed in panel C). Each trace represents the mean ± SD of three independent measurements (shaded area). C) Rifampicin induced a lag phase in *Sb*POR2b proteoliposome activity towards RS. Time trajectories of emission intensity are shown in the presence of varying rifampicin concentrations (0-200 μM). Reaction rates were extracted after the lag phase for all screening and dose-response experiments. Each trace represents the mean ± SD of triplicate measurements (shaded area). We note that the lag phase is only observed for the *Sb*POR2b isoform towards RS. It is not apparent using human POR in neither detergent micelles nor proteoliposomes. Deciphering the mechanism underlying the lag phase extends beyond the scope of this work, however, is worth investigation in future studies.

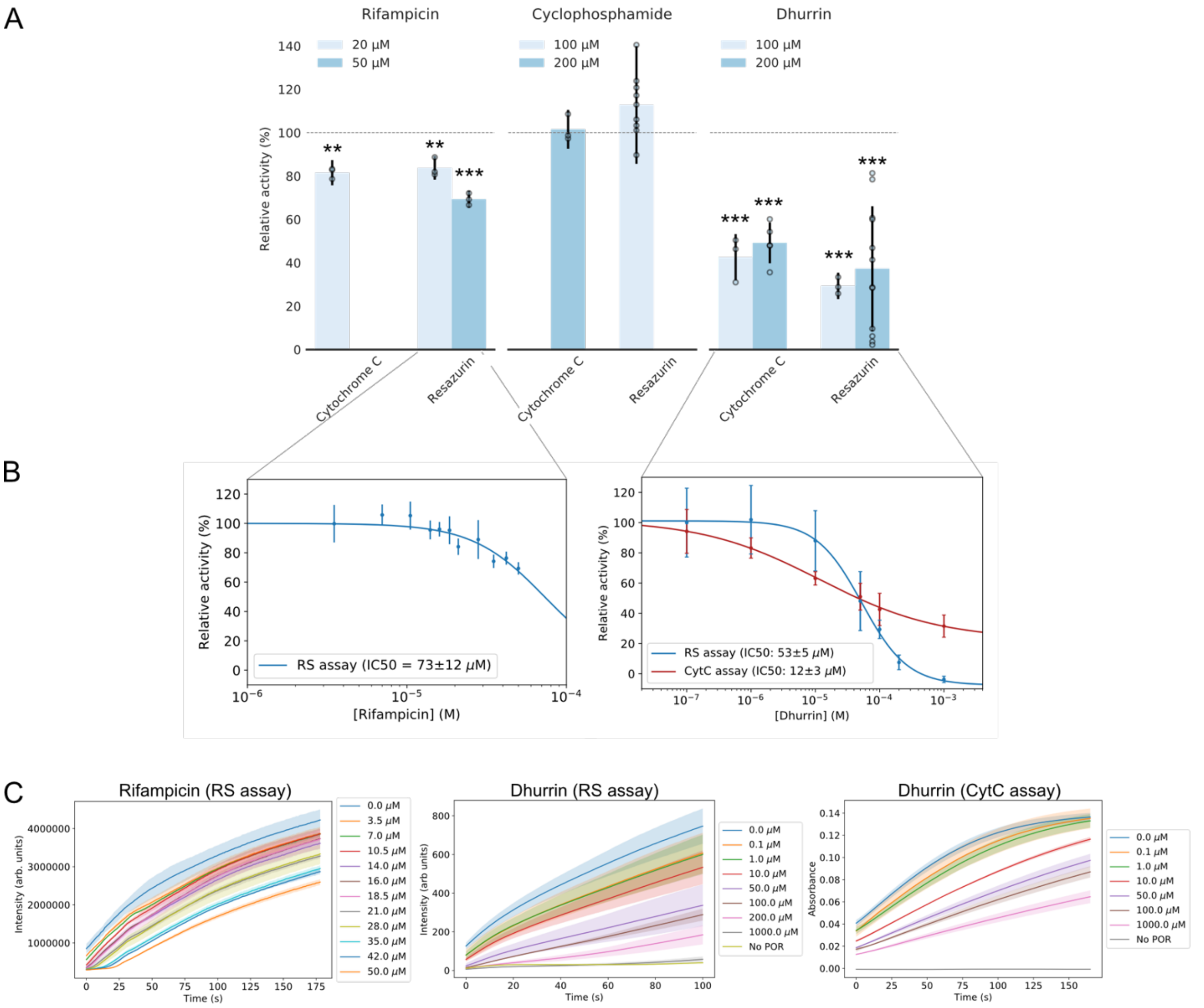

Supplementary Fig. 10. *In vitro* assays of small-molecule ligands on *Sb*POR2b in detergent micelles. A) Bar chart displaying POR activity normalized to controls in the Cyt*c* and RS assay, respectively. Error bars represent the mean ± SD of independent measurements normalized to controls with error propagation (n=3-12; see Supplementary Table 5 for exact value of n for each experimental condition). Overlapping data points appear shaded. The level of significance determined by one-way ANOVA and Tukey's HSD test correcting for multiple comparisons is marked by asterisk symbols (* p<0.05; ** p<0.01; *** p<0.005; see Supplementary methods for details). B) Dose-response curves of rifampicin (left) and dhurrin (right). IC50 values are depicted in the figure legend (low micromolar range). Error bars represent mean ± SD of three independent measurements normalized to controls with error propagation C) Raw activity traces of *Sb*POR2b underlying the dose-response curves. The linear region of each trace was used to quantify activity. For rifampicin traces, the activity was extracted after the lag phase according to Supplementary Fig 7. Each trace represents the mean±SD of three independent measurements (shaded area).

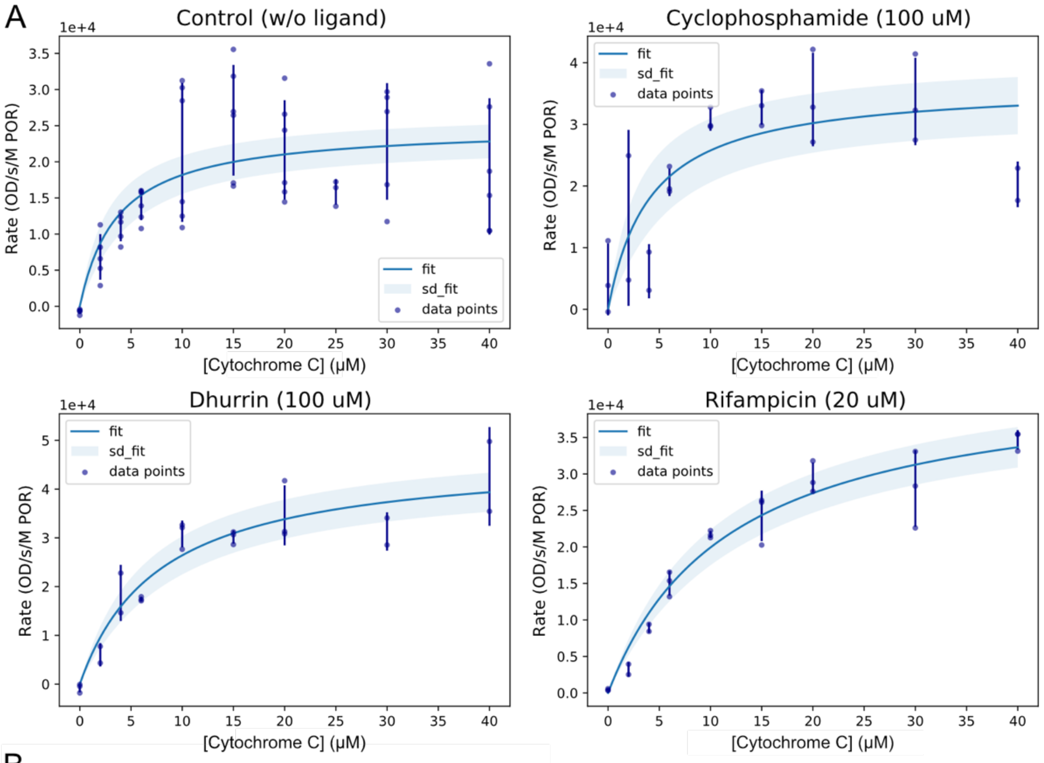

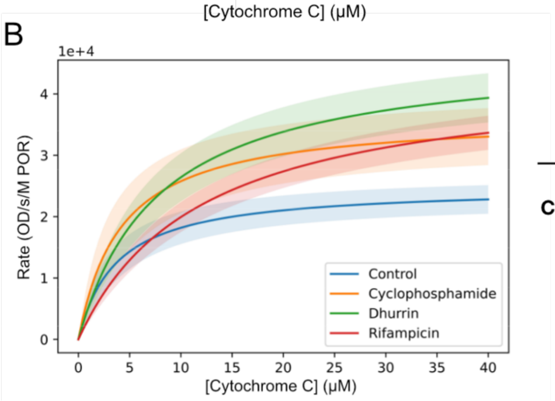

	K_M (μM)	V_{max} (OD/s/M POR)
**Control**	3.7±1.5	$(2.5\pm0.2)\cdot10^4$
**Cyclophosphamide**	4.2±2.1	$(3.6\pm0.5)\cdot10^4$
**Dhurrin**	7.8±2.1	$(4.7\pm0.4)\cdot10^4$
**Rifampicin**	12.0±2.2	$(4.4\pm0.3)\cdot10^4$

Supplementary Fig. 11. A) *In vitro* Michaelis-Menten kinetics displaying the effect of small molecule ligands on *Sb*POR2b activity of reducing Cyt*c* in liposomes. Error bars represent the mean ± SD of independent measurements (n=3 for cyclophosphamide, dhurrin and rifampicin; n=6 for POR w/o ligand except for 25 µM cyt*c* where n=3). Note, overlapping data points appear shaded. B) Overlay of Michaelis-Menten fits from panel A (left) and table of extracted K_M and V_{max} parameters (right) showing the effect of each ligand. All three ligands increase V_{max} as compared to control suggesting non-competitive binding, while dhurrin and rifampicin also affect K_M suggesting uncompetitive binding. Uncertainties of V_{max} and K_M are extracted from the standard error of the fit.

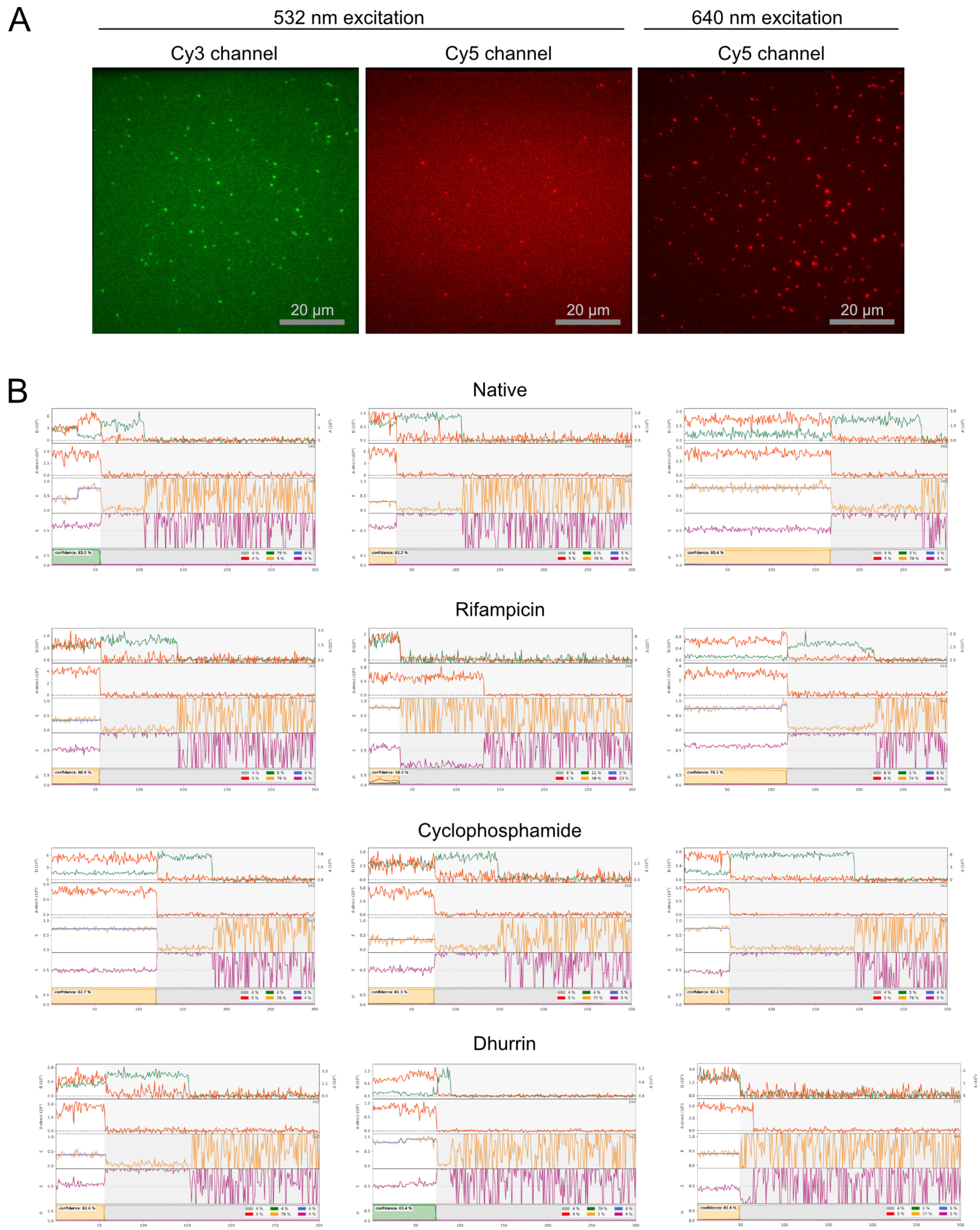

Supplementary Fig. 12. Representative raw TIRF microscope images and smFRET traces. A) Representative TIRF microscope images displaying one field-of-view (82x82 µm) of a surface with diffraction-limited, immobilized, dual-labelled *Sb*POR2b in nanodiscs excited at 532 nm and 640 nm, respectively. The images were acquired using emission filters optimized for Cy3 and Cy5 fluorophores (see Supplementary methods) and show one frame from movies recorded at 200 ms

temporal resolution. The number of recorded movies were 69, 26, 36 and 31 for native POR, rifampicin, cyclophosphamide and dhurrin, respectively. Data were collected from at least three different microscope coverslips for each experimental condition. B) Representative smFRET traces at each experimental condition. Every trace has four panels: The top panel displays donor (green) and acceptor (red) intensities at 532 nm excitation. The second panel displays acceptor intensity (red) at 640 nm excitation using ALEX. The third panel displays the FRET value (orange) calculated with calibration factors, and idealized FRET value determined from HMM fitting (blue). The fourth panel displays the donor-acceptor stoichiometry, which should ideally be 0.5 at a 1:1 donor-acceptor ratio. Some of the depicted traces display a static FRET value while others display transitions between long-lived equilibrium states. The x-axis represents frames (frame rate: 5 s^{-1}).

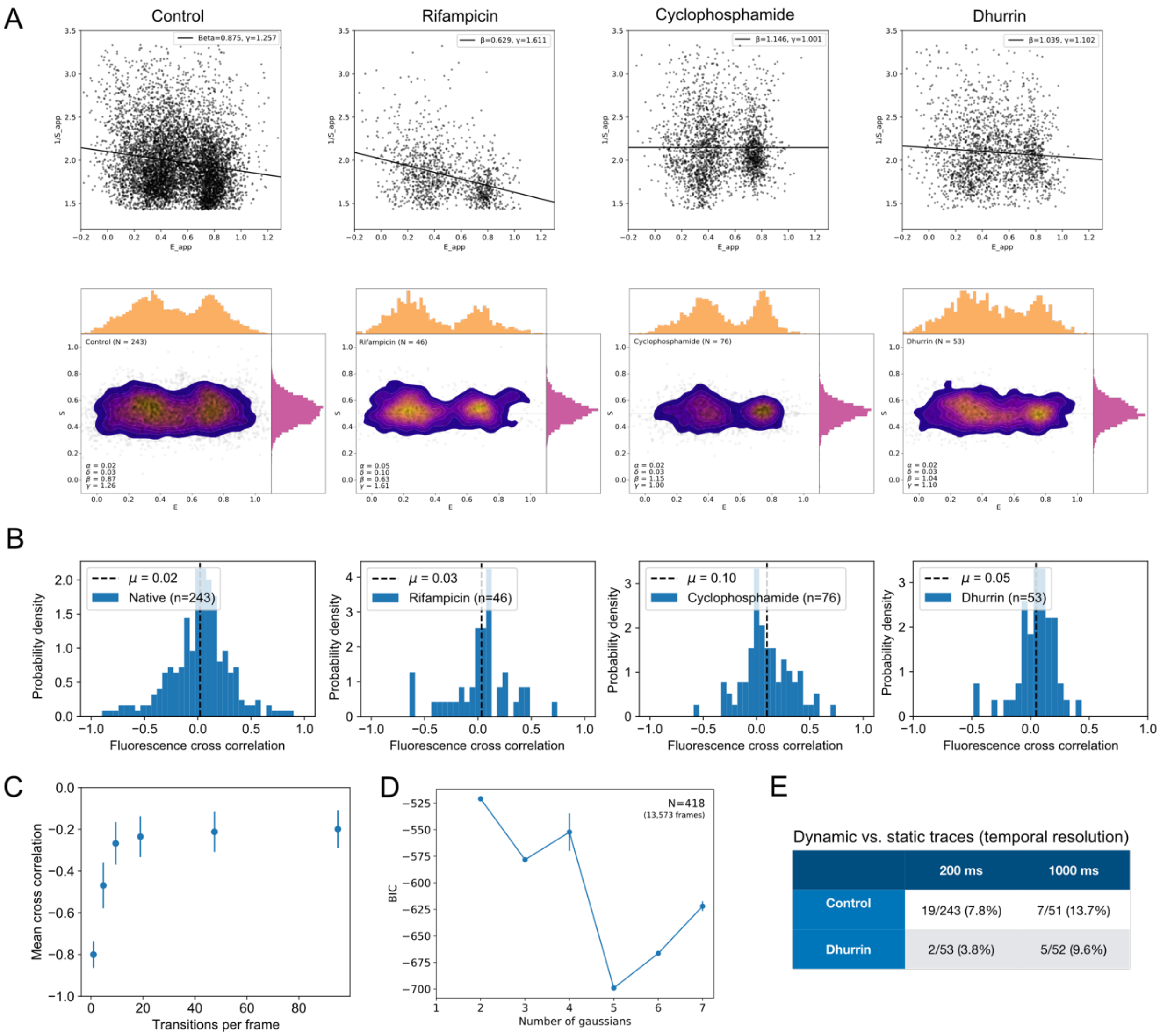

Supplementary Fig. 13. Correction factors, fluorescence cross correlation and BIC analysis of smFRET data. A) Determination of β and γ correction factors from global fitting of ALEX smFRET data following published methodology [6] (top), and 2D histograms displaying smFRET data after applying correction factors (bottom). B) Pearson correlation histograms of *Sb*POR2b smFRET data in the native form and in the presence of rifampicin, cyclophosphamide and dhurrin, respectively, displaying no autocorrelation in agreement with recent published data [7,8]. C) Simulation of smFRET traces displaying the mean cross correlation of simulated donor and acceptor intensities for varying temporal resolution in agreement with earlier studies of fast conformational transitions on GPCRs [9]. A two-state kinetic model with a 95% transition probability between each conformational state was used to simulate donor and acceptor trajectories without bleaching. 8% gaussian noise was added to each trace to mimic experimental uncertainty. The traces were subsequently binned using bin sizes varying from 1 to 100 to mimic various temporal resolutions. Increasing the bin size (i.e. average number of transitions per frame) results in loss of anticorrelation. Data display mean ± SD of 1024 simulated traces. D) Bayesian Information Criterion (BIC) scores of pooled *Sb*POR2b smFRET data (n=418 single molecules)

from fitting gaussian mixture models ranging from 2 to 7 gaussians. The lowest BIC score is obtained with a 5-state gaussian mixture model. Error bars represent mean ± SEM from bootstrapping (n=20 bootstrap iterations). E) Fraction of smFRET traces showing dynamic transitions as a function of temporal resolution. The fraction of traces showing transitions increases slightly when decreasing temporal resolution from 200 ms to 1 s, indicating that traces do not display an ensemble average but rather transitions between long-lived equilibrium states. The increase in observed transitions at 1 s as compared to 200 ms temporal resolution is caused by longer observation times allowing for more transitions to occur before photobleaching. A-B) N represents the number of single molecules at each experimental condition.

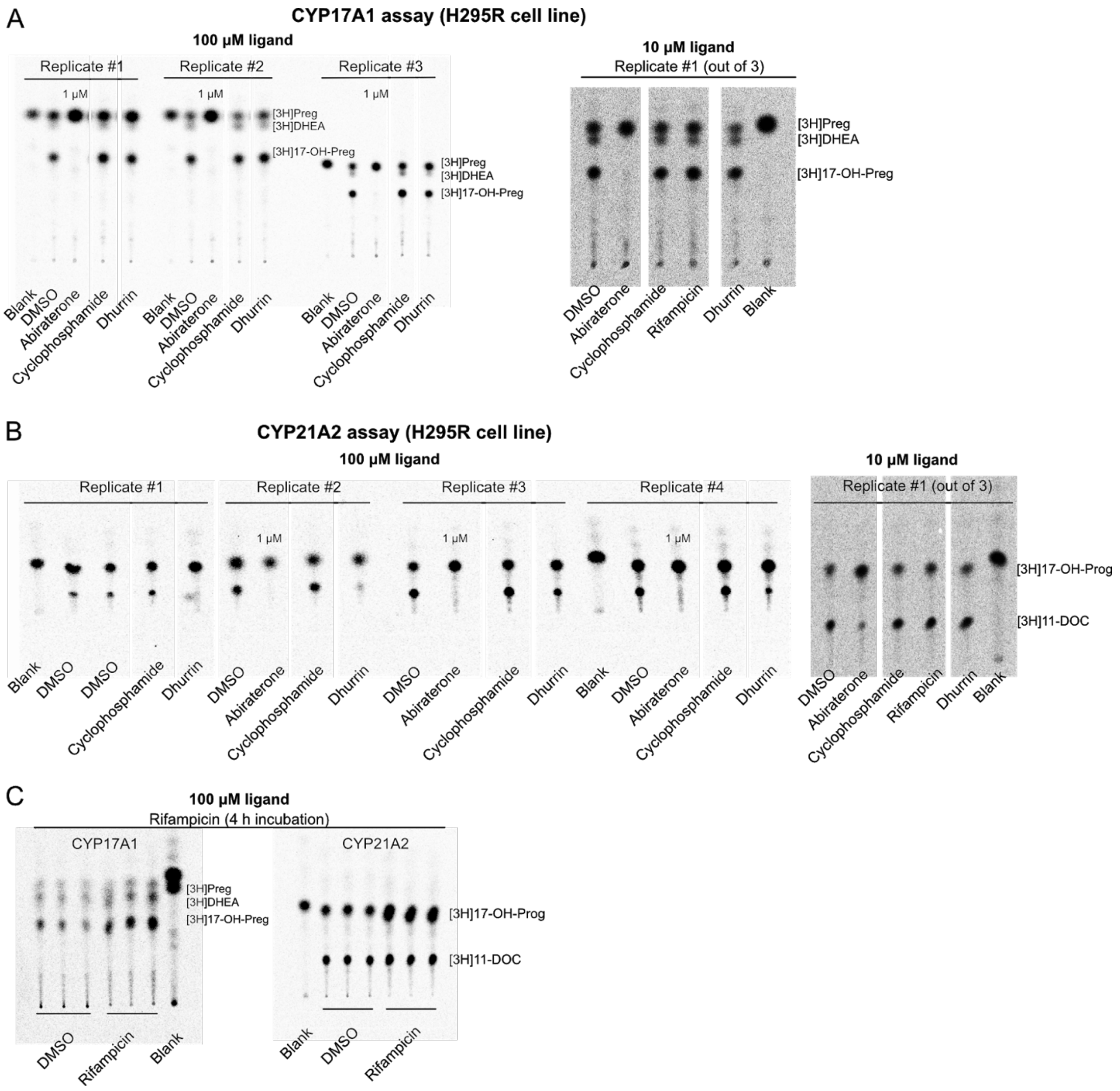

Supplementary Fig. 14. Raw TLC images of CYP17A1 activity assay (A) and CYP21A2 activity assay (B) in H295R cells at 100 µM and 10 µM ligand concentrations. Cells were incubated with ligand for 24 h before adding radiolabeled substrate and probing activity. Radiolabeled substrates and products were visualized on a PhosphorImager and quantified using Multi Gauge software. C) CYP17A1 and CYP21A2 assay at 100 µM rifampicin upon 4 h of incubation. A-C) Note, images are vertically sliced to juxtapose lanes that were non-adjacent in the original gels. Ligand effects are normalized to their respective blanks and DMSO controls before quantitative comparison between gels.

Protein	Site	SiteScore	Dscore	Volume (Å3)	Exposure	Enclosure	Residues defining the site
3ES9	Site II	1,06	0,98	584	0,56	0,79	97,101,104,105,214,217,218,220,221,224,225,228,235,240,241,242,243,244,245,357,360,361,375,381,382,384,385,387,388,446,447,448,449,450
	Site I	1,00	0,96	751	0,59	0,70	291,297,298,299,325,326,327,328,330,333,378,379,424,426,428,429,432,452,453,454,455,474,475,476,477,478,484,485,486,487,488,489,492,493,495,496,533,534,535,536,567,631,632,634,636,677,678,752,753
	Site III	0,93	0,95	273	0,73	0,61	198,199,200,201,202,203,204,205,215,218,219,222,225,226,229,230,382,383,404,406,407,408,409,410,411,412,413,416,417
	Site IV	0,84	0,74	158	0,63	0,62	290,291,292,293,294,302,304,470,541,545,569,570,571,572,573,574,575,576,577,578
	Site V	0,71	0,65	128	0,75	0,58	228,229,230,232,233,234,235,240,241,387,388,390,391,403
3QE2	Site I	1,03	0,98	2988	0,53	0,74	69,75,78,79,81,87,89,90,91,92,93,95,96,97,99,100,102,103,104,105,106,107,108,111,112,113,114,115,116,118,119,120,143,149,150,151,178,181,211,212,213,214,215,216,220,223,224,227,228,231,237,238,243,244,245,246,247,248,262,266,267,278,279,280,281,282,294,299,300,301,302,313,314,315,316,317,318,326,353,354,355,357,358,359,360,361,362,363,364,365,366,378,379,380,381,382,383,384,385,386,387,388,390,391,409,410,411,412,419,424,427,430,448,449,450,451,452,453,454,455,456,457,458,477,478,479,480,481,494,498,515,516,517,518,536,537,538,539,570,633,634,636,638,679,680,751,752,753
	Site II	1,00	0,98	689	0,55	0,70	90,143,144,145,146,147,148,149,150,153,179,180,181,182,183,184,318,319,322,458,466,516,517,518,519,520,521,522,523,524,525,526,527,546,549,550,553,628,630,631,635,636,637,639,640,642,643,647,666,669,670,672,674,675,676,677,678,751,752
	Site III	1,00	1,02	456	0,63	0,68	263,264,265,270,272,273,286,328,329,330,331,332,333,334,336,371,375,376,380,381,382,383,427,428,429,431,432,435,457,479,481,483,487,488,489,490,491,492,495,496,499,500,509,510,511,513,752
	Site V	0,65	0,49	74	0,72	0,55	310,311,312,313,314,315,316,317,318,465,469,470
	Site IV	0,60	0,53	78	0,71	0,55	293,294,295,296,297,572,574,575,576,577,578,579,580,581

Supplementary Table 1. Potential ligand binding sites on human POR in a compact conformation (PDB 3QE2) and rat POR in an extended conformation (PDB 3ES9) identified from SiteMap analysis. Sites I-III display SiteScore and Dscore values indicating that ligands may bind to these sites with submicromolar affinity, while exposure and enclosure scores of sites I-II are close to the average scores of tight-binding sites (0.49 exposure and 0.78 enclosure according to SiteMap manual). Note, Sites I-V on human POR do not completely align with Sites I-V on rat POR.

Protein	Ligand	Site	Gscore	Emodel	Residues within 5 Å from the ligand
3ES9	Cyclophosphamide	Site I	-5.0	-40.5	E297, R298, H299, A475, V476, E477, Y478, R567, FAD, NADPH
		Site II	-4.6	-32.2	-
		Site V	-4.4	-36.7	-
		Site IV	-4.2	-30.6	-
		Site III	-4.1	-32.3	-
	Dhurrin	Site I	-7.0	-67.4	R298, V474, A475, V476, E477, Y478, T535, R567, W677, S678
		Site II	-5.7	-56.8	-
		Site V	-5.2	-54.3	-
		Site III	-5.1	-47.8	-
		Site IV	-4.7	-44.9	-
	Rifampicin	Site I	-4.6	-51.2	E297-L300 (ERHL), V474-E479 (VAVEYE), R567, R597, K602, S678
3QE2	Cyclophosphamide	Site Ia	-5.2	-45.3	V477, V479, P536-**G539*** (PGTG), G633, D634, M638, W679, S680, FAD, NADPH
		Site Ib	-5.0	-45.3	Q90, T91, Q92, D150, A281, K282, A324, Y326, Q455, A456, Y458, F515, R517
		Site III	-4.5	-35.7	-
		Site IV	-4.1	-31.7	-
		Site II	-3.9	-34.2	-
		Site V	-3.0	-22.4	-
	Dhurrin	Site Ib	-6.7	-68.3	Y87, S89, Q90, G92, E95, E118, E119, N151, M266, A281, K282, Y326, K360, Y376, Y377, R453, L454, Q455
		Site Ia	-6.3	-69.0	V477, G537, T538, **G539***, R570, G633, D634, R636, W679, S680, FAD, NADPH
		Site III	-6.2	-67.1	-
		Site II	-5.7	-60.5	-
		Site V	-5.6	-47.3	-
		Site IV	-5.3	-46.6	-
	Rifampicin	Site Ia	-6.1	-83.6	R301, R427, V479, P536, T538, **G539***, R570, **R600***, G633-N637 (GDARN), W679, S680, FAD, NADPH

* Residues associated with POR deficiency

Supplementary Table 2. Binding energies (Gscore; kcal/mol) and Emodel scores of small-molecule ligands on human POR in a compact conformation (PDB 3QE2) and rat POR in an extended conformation (PDB 3ES9) predicted from computational docking simulations. Only the lowest energy score of each ligand in each site is considered. Amino acid residues within 5 Å from the docked ligands in Sites Ia and Ib of human POR and Site I of rat POR are shown. Human POR residues associated with POR deficiency are marked as bold. G539R and R600W are both found in patients with POR deficiency and cause disorder of sexual development due to low production of sex steroids [2,5].

	POR isoform	Reconstitution	Assay	Ligand	[Drug] (uM)	Relative activity	Standard deviation	Standard error of the mean	nobs (drug)	nobs (control)	n_dates
					Fig 2						
0	hPOR	Liposomes	Cytochrome C	Cyclophosphamide	10.0	106.5	5.8	2.4	6	6	2
1	hPOR	Liposomes	Cytochrome C	Dhurrin	10.0	110.1	11.0	5.3	4	6	2
2	hPOR	Liposomes	Cytochrome C	Rifampicin	10.0	112.6	7.5	3.2	5	6	2
3	hPOR	Liposomes	Cytochrome C	Cyclophosphamide	100.0	119.3	14.4	8.6	2	3	1
4	hPOR	Liposomes	Cytochrome C	Dhurrin	100.0	48.4	9.0	5.2	3	3	1
5	hPOR	Liposomes	Cytochrome C	Rifampicin	100.0	14.0	5.1	2.9	3	3	1
6	hPOR	Liposomes	MTT	Cyclophosphamide	10.0	100.3	6.2	4.0	2	3	1
7	hPOR	Liposomes	MTT	Dhurrin	10.0	87.7	6.6	3.8	3	3	1
8	hPOR	Liposomes	MTT	Rifampicin	10.0	95.7	13.2	7.6	3	3	1
9	hPOR	Liposomes	MTT	Cyclophosphamide	100.0	99.6	3.4	2.0	3	3	1
10	hPOR	Liposomes	MTT	Dhurrin	100.0	95.9	2.5	1.5	3	3	1
11	hPOR	Liposomes	MTT	Rifampicin	100.0	122.3	2.6	1.5	3	3	1
12	hPOR	Liposomes	Resazurin	Cyclophosphamide	10.0	115.0	6.6	2.7	6	6	2
13	hPOR	Liposomes	Resazurin	Dhurrin	10.0	105.2	9.8	4.0	6	6	2
14	hPOR	Liposomes	Resazurin	Rifampicin	10.0	132.4	5.2	2.1	6	6	2
15	hPOR	Liposomes	Resazurin	Cyclophosphamide	100.0	103.6	10.1	5.8	3	3	1
16	hPOR	Liposomes	Resazurin	Dhurrin	100.0	81.7	5.8	3.3	3	3	1
17	hPOR	Liposomes	Resazurin	Rifampicin	100.0	312.1	16.0	9.2	3	3	1
					Fig S1						
18	hPOR	Liposomes	Cytochrome C	Amiodarone	10.0	95.6	9.8	4.0	6	6	2
19	hPOR	Liposomes	Cytochrome C	Cimetidine	10.0	110.2	10.5	4.6	5	6	2
20	hPOR	Liposomes	Cytochrome C	Mitomycin C	10.0	101.1	13.6	7.9	3	3	1
21	hPOR	Liposomes	Cytochrome C	Ritonavir	10.0	108.0	6.1	2.6	5	6	2
22	hPOR	Liposomes	Cytochrome C	Warfarin	10.0	102.3	11.3	6.5	3	3	1
23	hPOR	Liposomes	Cytochrome C	Amiodarone	100.0	106.0	12.6	7.3	3	3	1
24	hPOR	Liposomes	Cytochrome C	Cimetidine	100.0	117.1	19.7	11.4	3	3	1
25	hPOR	Liposomes	Cytochrome C	Ritonavir	100.0	118.0	16.3	9.4	3	3	1
26	hPOR	Liposomes	MTT	Amiodarone	10.0	85.1	9.7	5.6	3	3	1
27	hPOR	Liposomes	MTT	Cimetidine	10.0	96.9	8.5	4.9	3	3	1
28	hPOR	Liposomes	MTT	Mitomycin C	10.0	110.4	16.0	9.3	3	2	1
29	hPOR	Liposomes	MTT	Ritonavir	10.0	88.4	8.2	5.6	2	3	1
30	hPOR	Liposomes	MTT	Warfarin	10.0	118.4	12.7	7.3	3	2	1
31	hPOR	Liposomes	Resazurin	Amiodarone	10.0	102.2	7.2	3.0	6	6	2
32	hPOR	Liposomes	Resazurin	Cimetidine	10.0	107.0	11.1	4.5	6	6	2
33	hPOR	Liposomes	Resazurin	Mitomycin C	10.0	53.7	5.9	3.4	3	3	1
34	hPOR	Liposomes	Resazurin	Ritonavir	10.0	96.9	6.8	2.8	6	6	2
35	hPOR	Liposomes	Resazurin	Warfarin	10.0	96.2	5.4	3.1	3	3	1
36	hPOR	Liposomes	Resazurin	Amiodarone	100.0	101.5	9.3	5.4	3	3	1
37	hPOR	Liposomes	Resazurin	Cimetidine	100.0	112.0	9.5	5.5	3	3	1
38	hPOR	Liposomes	Resazurin	Ritonavir	100.0	95.9	7.8	4.5	3	3	1
					Fig 3						
39	SbPOR2b	Liposomes	Cytochrome C	Cyclophosphamide	10.0	100.1	14.1	4.8	6	12	2
40	SbPOR2b	Liposomes	Cytochrome C	Dhurrin	10.0	127.6	23.7	12.6	3	6	1
41	SbPOR2b	Liposomes	Cytochrome C	Rifampicin	10.0	140.3	18.5	9.0	3	6	1
42	SbPOR2b	Liposomes	Cytochrome C	Cyclophosphamide	100.0	103.9	16.0	5.7	6	12	2
43	SbPOR2b	Liposomes	Cytochrome C	Dhurrin	100.0	155.5	17.1	7.6	3	6	1
44	SbPOR2b	Liposomes	Cytochrome C	Rifampicin	100.0	178.8	34.7	18.6	3	6	1
45	SbPOR2b	Liposomes	Resazurin	Cyclophosphamide	10.0	98.1	21.0	10.3	3	6	1
46	SbPOR2b	Liposomes	Resazurin	Dhurrin	10.0	127.5	41.4	9.3	12	21	3
47	SbPOR2b	Liposomes	Resazurin	Rifampicin	10.0	101.7	15.5	6.3	3	6	1
48	SbPOR2b	Liposomes	Resazurin	Cyclophosphamide	100.0	108.3	19.4	7.3	5	9	2
49	SbPOR2b	Liposomes	Resazurin	Dhurrin	100.0	235.6	96.1	32.4	6	9	1
50	SbPOR2b	Liposomes	Resazurin	Rifampicin	100.0	85.0	13.2	5.5	3	6	1

Supplementary Table 3. Experimental details of *in vitro* activity assays on hPOR and *Sb*POR2b reconstituted in proteoliposomes including number of replicates (nobs) for both ligand and DMSO controls. Associated p-values were calculated based on one-way ANOVA and Tukey's HSD test correcting for multiple comparisons. The levels of significance are marked by asterisk symbols on the corresponding bar charts (see Fig 1-3, Supplementary Fig 1 and Supplementary methods "Statistical analysis of in vitro activity data" for details).

	POR isoform	Reconstitution	Assay	Ligand	[Drug] (uM)	Relative activity	Standard deviation	Standard error of the mean	nobs (drug)	nobs (control)	n_dates
	Fig 5A										
0	hPOR	Cells (NCI-H295R)	CYP17 (17,20-lyase)	Cyclophosphamide	10.0	110.7	12.9	7.5	3	3	3
1	hPOR	Cells (NCI-H295R)	CYP17 (17,20-lyase)	Dhurrin	10.0	106.6	10.5	6.1	3	3	3
2	hPOR	Cells (NCI-H295R)	CYP17 (17,20-lyase)	Rifampicin	10.0	84.7	12.9	7.4	3	3	3
3	hPOR	Cells (NCI-H295R)	CYP17 (17,20-lyase)	Cyclophosphamide	100.0	132.3	7.9	4.6	3	3	3
4	hPOR	Cells (NCI-H295R)	CYP17 (17,20-lyase)	Dhurrin	100.0	79.5	6.9	4.0	3	3	3
5	hPOR	Cells (NCI-H295R)	CYP17 (17,20-lyase)	Rifampicin	100.0	90.7	11.4	6.6	3	3	3
6	hPOR	Cells (NCI-H295R)	CYP17 (17-OHase)	Cyclophosphamide	10.0	106.2	5.8	3.3	3	3	3
7	hPOR	Cells (NCI-H295R)	CYP17 (17-OHase)	Dhurrin	10.0	107.6	5.6	3.3	3	3	3
8	hPOR	Cells (NCI-H295R)	CYP17 (17-OHase)	Rifampicin	10.0	100.9	2.3	1.3	3	3	3
9	hPOR	Cells (NCI-H295R)	CYP17 (17-OHase)	Cyclophosphamide	100.0	111.1	1.0	0.6	3	3	3
10	hPOR	Cells (NCI-H295R)	CYP17 (17-OHase)	Dhurrin	100.0	90.6	12.0	6.9	3	3	3
11	hPOR	Cells (NCI-H295R)	CYP17 (17-OHase)	Rifampicin	100.0	111.1	13.1	7.6	3	3	3
12	hPOR	Cells (NCI-H295R)	CYP21 (21-OHase)	Cyclophosphamide	10.0	104.1	0.8	0.4	3	3	3
13	hPOR	Cells (NCI-H295R)	CYP21 (21-OHase)	Dhurrin	10.0	103.1	5.3	3.1	3	3	3
14	hPOR	Cells (NCI-H295R)	CYP21 (21-OHase)	Rifampicin	10.0	96.9	3.1	1.8	3	3	3
15	hPOR	Cells (NCI-H295R)	CYP21 (21-OHase)	Cyclophosphamide	100.0	113.1	10.9	5.4	4	4	4
16	hPOR	Cells (NCI-H295R)	CYP21 (21-OHase)	Dhurrin	100.0	44.0	10.8	5.4	4	4	4
17	hPOR	Cells (NCI-H295R)	CYP21 (21-OHase)	Rifampicin	100.0	73.0	4.1	2.4	3	3	3
18	hPOR	Cells (NCI-H295R)	MTT cell viability	Cyclophosphamide	10.0	87.9	3.5	1.7	3	8	1
19	hPOR	Cells (NCI-H295R)	MTT cell viability	Dhurrin	10.0	93.6	4.8	2.5	3	8	1
20	hPOR	Cells (NCI-H295R)	MTT cell viability	Rifampicin	10.0	94.0	3.3	1.5	3	8	1
21	hPOR	Cells (NCI-H295R)	MTT cell viability	Cyclophosphamide	100.0	81.4	2.6	1.1	3	8	1
22	hPOR	Cells (NCI-H295R)	MTT cell viability	Dhurrin	100.0	88.1	3.4	1.6	3	8	1
23	hPOR	Cells (NCI-H295R)	MTT cell viability	Rifampicin	100.0	115.7	3.4	1.3	3	8	1
	Fig 5B										
24	hPOR	Cells (NCI-H295R)	CYP17 (17,20-lyase)	Abiraterone	1.0	13.9	14.2	8.2	3	3	3
25	hPOR	Cells (NCI-H295R)	CYP17 (17,20-lyase)	Abiraterone	10.0	37.3	19.5	11.2	3	3	3
26	hPOR	Cells (NCI-H295R)	CYP17 (17-OHase)	Abiraterone	1.0	3.7	2.9	1.6	3	3	3
27	hPOR	Cells (NCI-H295R)	CYP17 (17-OHase)	Abiraterone	10.0	8.8	3.9	2.3	3	3	3
28	hPOR	Cells (NCI-H295R)	CYP21 (21-OHase)	Abiraterone	1.0	11.6	2.6	1.5	3	3	3
29	hPOR	Cells (NCI-H295R)	CYP21 (21-OHase)	Abiraterone	10.0	14.1	4.7	2.7	3	3	3
	Fig 5C										
30	hPOR	Microsomes (JEG3)	19,00 CYP	Cyclophosphamide	10	102.7	22.0	11.1	3	4	1
31	hPOR	Microsomes (JEG3)	19,00 CYP	Dhurrin	10	101.4	22.0	11.2	3	4	1
32	hPOR	Microsomes (JEG3)	19,00 CYP	Rifampicin	10	80.7	19.9	10.5	3	4	1
33	hPOR	Microsomes (JEG3)	19,00 CYP	Rifampicin	50	44.8	20.0	11.6	3	3	1
34	hPOR	Microsomes (JEG3)	19,00 CYP	Cyclophosphamide	100	108.5	21.5	12.4	3	3	1
35	hPOR	Microsomes (JEG3)	19,00 CYP	Dhurrin	100	94.0	25.5	14.7	3	3	1
36	hPOR	Microsomes (JEG3)	19,00 CYP	Rifampicin	100	31.9	10.6	6.1	3	3	1

Supplementary Table 4. Experimental details of CYP17A1, CYP21A2 and MTT cell viability assays on NCI-H295R cell line and CYP19A1 assay on microsomes from JEG3 cells including number of replicates (nobs) for both ligand and DMSO controls. Associated p-values were calculated based on one-way ANOVA and Tukey’s HSD test correcting for multiple comparisons. The levels of significance are marked by asterisk symbols on the corresponding bar charts (see Fig 5 and Supplementary methods “CYP17A1 and CYP21A2 activity assay in H295R cells”, “MTT cell viability assay”, “CYP19A1 activity assay in JEG3 microsomes” and “Statistical analysis of in vitro activity data” for details).

	POR isoform	Reconstitution	Assay	Drug	[Drug] (uM)	Relative activity	Standard deviation	Standard error of the mean	nobs (drug)	nobs (control)	n_dates
0	SbPOR2b	Detergent	Cytochrome C	Rifampicin	20.0	81.6	5.8	3.4	3	3	1
1	SbPOR2b	Detergent	Cytochrome C	Dhurrin	100.0	42.6	10.6	6.1	3	3	1
2	SbPOR2b	Detergent	Cytochrome C	Cyclophosphamide	200.0	101.6	9.0	5.2	3	3	1
3	SbPOR2b	Detergent	Cytochrome C	Dhurrin	200.0	49.2	9.4	4.2	5	5	2
4	SbPOR2b	Detergent	Resazurin	Rifampicin	20.0	83.8	5.4	3.1	3	3	1
5	SbPOR2b	Detergent	Resazurin	Rifampicin	50.0	69.3	3.9	2.2	3	3	1
6	SbPOR2b	Detergent	Resazurin	Cyclophosphamide	100.0	112.8	27.2	9.1	9	9	3
7	SbPOR2b	Detergent	Resazurin	Dhurrin	100.0	29.4	6.1	3.5	3	3	1
8	SbPOR2b	Detergent	Resazurin	Dhurrin	200.0	37.3	28.8	8.3	12	19	6

Supplementary Table 5. Experimental details of *in vitro* activity assays on *Sb*POR2b reconstituted in detergent including number of replicates (nobs) for both ligand and DMSO controls. Associated p-values were calculated based on one-way ANOVA and Tukey's HSD test correcting for multiple comparisons. The levels of significance are marked by asterisk symbols on the corresponding bar charts (see Supplementary Fig 10 and Supplementary methods "Statistical analysis of in vitro activity data" for details).

References

1. Wang, S.-L., Han, J.-F., He, X.-Y., Wang, X.-R. & Hong, J.-Y. Genetic variation of human cytochrome p450 reductase as a potential biomarker for mitomycin C-induced cytotoxicity. *Drug Metab. Dispos.* **35,** 176–179 (2007).
2. Pandey, A. V. & Flück, C. E. NADPH P450 oxidoreductase: structure, function, and pathology of diseases. *Pharmacol. Ther.* **138,** 229–254 (2013).
3. Nicolo, C., Flück, C. E., Mullis, P. E. & Pandey, A. V. Restoration of mutant cytochrome P450 reductase activity by external flavin. *Mol. Cell. Endocrinol.* **321,** 245–252 (2010).
4. Burkhard, F. Z., Parween, S., Udhane, S. S., Flück, C. E. & Pandey, A. V. P450 Oxidoreductase deficiency: Analysis of mutations and polymorphisms. *J. Steroid Biochem. Mol. Biol.* **165,** 38–50 (2017).
5. Huang, N., Agrawal, V., Giacomini, K. M. & Miller, W. L. Genetics of P450 oxidoreductase: sequence variation in 842 individuals of four ethnicities and activities of 15 missense mutations. *Proc. Natl. Acad. Sci. USA* **105,** 1733–1738 (2008).
6. Hellenkamp, B. *et al.* Precision and accuracy of single-molecule FRET measurements-a multi-laboratory benchmark study. *Nat. Methods* **15,** 669–676 (2018).
7. Frances, O. *et al.* A well-balanced preexisting equilibrium governs electron flux efficiency of a multidomain diflavin reductase. *Biophys. J.* **108,** 1527–1536 (2015).
8. Laursen, T. *et al.* Single molecule activity measurements of cytochrome P450 oxidoreductase reveal the existence of two discrete functional states. *ACS Chem. Biol.* **9,** 630–634 (2014).
9. Gregorio, G. G. *et al.* Single-molecule analysis of ligand efficacy in β2AR-G-protein activation. *Nature* **547,** 68–73 (2017).
