# Supplementary material for: Biased cytochrome P450-mediated metabolism via small-molecule ligands binding P450 oxidoreductase
Source: Nat Commun. 2021 Apr 15;12:2260. doi: 10.1038/s41467-021-22562-w (PMC8050233; doi:10.1038/s41467-021-22562-w)
Supplement: Supplementary file 7 — Description of Additional Supplementary Files [file 41467_2021_22562_MOESM7_ESM.docx]

Description of additional supplementary information

Title: Supplementary Movie 1.

Description: 1000 ns MD simulation of cyclophosphamide binding to the closed form of human POR (PDB 3QE2) displayed in the same view as the figures in the text. Cartoon representation displays the NADPH-binding domain (brown), FAD-binding domain (orange) and FMN-binding domain (cyan). Ligands are displayed in green, while cofactors are in yellow. The binding mode is stable after an initial equilibration phase (see Cα RMSD in Supplementary Fig 5).

Title: Supplementary Movie 2.

Description: 1000 ns MD simulation of dhurrin binding to the closed form of human POR (PDB 3QE2) displayed in the same view as the figures in the text. Cartoon representation displays the NADPH-binding domain (brown), FAD-binding domain (orange) and FMN-binding domain (cyan). Ligands are displayed in green, while cofactors are in yellow. The binding mode is stable after an initial equilibration phase (see Cα RMSD in Supplementary Fig 5).

Title: Supplementary Movie 3.

Description: 1000 ns MD simulation of rifampicin binding to the closed form of human POR (PDB 3QE2) displayed in the same view as the figures in the text. Cartoon representation displays the NADPH-binding domain (brown), FAD-binding domain (orange) and FMN-binding domain (cyan). Ligands are displayed in green, while cofactors are in yellow. The binding mode is stable after an initial equilibration phase (see Cα RMSD in Supplementary Fig 5).

Title: Supplementary Movie 4.

Description: Selected frames from the MD simulations of cyclophosphamide, dhurrin and rifampicin binding to the closed form of POR (3EQ2). For each simulation the frames after 100, 200, …, 1000 ns are displayed first with the same perspective as the figures in the text and subsequently zoomed in showing the binding of the ligands in the binding site. Protein displayed as cartoon representations coloured continuously from blue at the N-terminal to red at the C-terminal. Cofactors and ligands are shown as stick models coloured according to atom types, except that C-atoms in the cofactors are cyan and in ligands are green.
