# Supplementary material for: Biased cytochrome P450-mediated metabolism via small-molecule ligands binding P450 oxidoreductase
Source: Nat Commun. 2021 Apr 15;12:2260. doi: 10.1038/s41467-021-22562-w (PMC8050233; doi:10.1038/s41467-021-22562-w)
Supplement: Supplementary file 8 — Reporting Summary [file 41467_2021_22562_MOESM8_ESM.pdf]

nature research

Corresponding author(s): Nikos Hatzakis

Last updated by author(s): Feb 26, 2021

Reporting Summary

Nature Research wishes to improve the reproducibility of the work that we publish. This form provides structure for consistency and transparency in reporting. For further information on Nature Research policies, see our Editorial Policies and the Editorial Policy Checklist.

Statistics

For all statistical analyses, confirm that the following items are present in the figure legend, table legend, main text, or Methods section.

n/a	Confirmed	
☐	☒	The exact sample size (*n*) for each experimental group/condition, given as a discrete number and unit of measurement
☐	☒	A statement on whether measurements were taken from distinct samples or whether the same sample was measured repeatedly
☐	☒	The statistical test(s) used AND whether they are one- or two-sided<br>*Only common tests should be described solely by name; describe more complex techniques in the Methods section.*
☐	☒	A description of all covariates tested
☐	☒	A description of any assumptions or corrections, such as tests of normality and adjustment for multiple comparisons
☐	☒	A full description of the statistical parameters including central tendency (e.g. means) or other basic estimates (e.g. regression coefficient) AND variation (e.g. standard deviation) or associated estimates of uncertainty (e.g. confidence intervals)
☒	☐	For null hypothesis testing, the test statistic (e.g. *F*, *t*, *r*) with confidence intervals, effect sizes, degrees of freedom and *P* value noted<br>*Give P values as exact values whenever suitable.*
☐	☒	For Bayesian analysis, information on the choice of priors and Markov chain Monte Carlo settings
☐	☒	For hierarchical and complex designs, identification of the appropriate level for tests and full reporting of outcomes
☒	☐	Estimates of effect sizes (e.g. Cohen's *d*, Pearson's *r*), indicating how they were calculated

Our web collection on statistics for biologists contains articles on many of the points above.

Software and code

Policy information about availability of computer code

Data collection: Olympus cellSens software (v2.2) was used to collect smFRET data from a TIRF micrsocope. Multi Gauge software was used to collect and quantify TLC silicagel images on a FujiFilm PhorporImager (Fuji FLA-7000).

Data analysis: The open-source software DeepFRET was used for smFRET data analysis (doi 10.7554/eLife.60404). Commercial softwares Glide and Desmond were used for computational docking and MD simulations, respectively. Statistical tests and data analysis were scripted in Python using standard open-source libraries (e.g. Numpy, Scipy, Pandas, Matplotlib, Statsmodels). All relevant softwares are cited in the methods section.

For manuscripts utilizing custom algorithms or software that are central to the research but not yet described in published literature, software must be made available to editors and reviewers. We strongly encourage code deposition in a community repository (e.g. GitHub). See the Nature Research guidelines for submitting code & software for further information.

Data

Policy information about availability of data

All manuscripts must include a data availability statement. This statement should provide the following information, where applicable:

- Accession codes, unique identifiers, or web links for publicly available datasets
- A list of figures that have associated raw data
- A description of any restrictions on data availability

All relevant data are displayed in the main figures or the supplementary materials. Source data underlying main and supplementary figures are deposited at UCPH ERDA server accessible via the link: https://sid.erda.dk/sharelink/EpwrxUSWOe

Field-specific reporting

Please select the one below that is the best fit for your research. If you are not sure, read the appropriate sections before making your selection.

☒ Life sciences ☐ Behavioural & social sciences ☐ Ecological, evolutionary & environmental sciences

For a reference copy of the document with all sections, see nature.com/documents/nr-reporting-summary-flat.pdf

Life sciences study design

All studies must disclose on these points even when the disclosure is negative.

Sample size	All data presented in the study are derived from biological replicates (n=2-12). Exact values of n for each experiment are displayed in supplementary tables 2-4.
Data exclusions	The selection/exclusion of smFRET traces from raw microscope images was performed using the deep neural network model implemented in DeepFRET (see doi 10.7554/eLife.60404). The number of recorded movies and the corresponding number of selected single molecule FRET traces are explicitly stated in the manuscript. No further data were excluded from analysis.
Replication	All attempts at replication were succesful. Error bars and statistical tests are defined in all relevant figure legends and supplementary methods.
Randomization	Not relevant when using biological replicates of cells and proteins.
Blinding	Not relevant when using biological replicates of cells and proteins.

Reporting for specific materials, systems and methods

We require information from authors about some types of materials, experimental systems and methods used in many studies. Here, indicate whether each material, system or method listed is relevant to your study. If you are not sure if a list item applies to your research, read the appropriate section before selecting a response.

Materials & experimental systems

n/a	Involved in the study	
☒	☐	Antibodies
☐	☒	Eukaryotic cell lines
☒	☐	Palaeontology and archaeology
☒	☐	Animals and other organisms
☒	☐	Human research participants
☒	☐	Clinical data
☒	☐	Dual use research of concern

Methods

n/a	Involved in the study	
☒	☐	ChIP-seq
☒	☐	Flow cytometry
☒	☐	MRI-based neuroimaging

Eukaryotic cell lines

Policy information about cell lines

Cell line source(s)	Human adrenal carcinoma cell line (NCI-H295R) and human placental JEG3 cell line was purchased from American Type Culture Collection (ATCC: CRL-2128 and HTB-36TM, respectively)
Authentication	Cell lines were not authenticated after purchasing from ATCC.
Mycoplasma contamination	Cell lines were not tested for mycoplasma contamination after purchasing from ATCC.
Commonly misidentified lines (See ICLAC register)	No commonly misidentified cell lines are registered in the ICLAC register.
